# An exploratory randomised trial investigating feasibility, potential impact and cost effectiveness of link workers for people living with multimorbidity attending general practices in deprived urban communities

**DOI:** 10.1186/s12875-024-02482-6

**Published:** 2024-06-28

**Authors:** Bridget Kiely, Anna Hobbins, Fiona Boland, Barbara Clyne, Emer Galvin, Vivienne Byers, Sonali Loomba, Patrick O’Donnell, Deirdre Connolly, Eamon O.’ Shea, Susan M. Smith

**Affiliations:** 1 Department of General Practice, Clinical Research Fellow, Royal College of Surgeons in Ireland University of Medicine and Health Sciences, 123 St Stephens Green, Dublin 2, Dublin, Ireland; 2 Centre for Research in Medical Devices (CÚRAM, RC/2073_P2) and Health Economics and Policy Analysis Centre, University of Galway, SFI 13, Galway, Ireland; 3 Data Science Centre, Royal College of Surgeons in Ireland University of Medicine and Health Sciences, 123 St Stephens Green, Dublin 2, Dublin, Ireland; 4 Department of Public Health and Epidemiology, Royal College of Surgeons in Ireland University of Medicine and Health Sciences, 123 St Stephens Green, Dublin 2, Dublin, Ireland; 5 Royal College of Surgeons in Ireland University of Medicine and Health Sciences, 123 St Stephens Green, Dublin 2, Dublin, Ireland; 6 Environment Sustainability and Health Institute, Technological University Dublin, Dublin, Ireland; 7 Graduate Entry Medical School, University of Limerick, Limerick, Ireland; 8 Discipline of Occupational Therapy, Trinity College, Dublin, Ireland; 9 School of Business and Economics, University of Galway, Galway, Ireland; 10 Discipline of Public Health and Primary Care, Trinity College, Dublin, Ireland

**Keywords:** Primary care, General practice, Multimorbidity, Social prescribing, Pragmatic clinical trial, Cost analysis

## Abstract

**Background:**

Social prescribing link workers are non-health or social care professionals who connect people with psychosocial needs to non-clinical community supports. They are being implemented widely, but there is limited evidence for appropriate target populations or cost effectiveness.

This study aimed to explore the feasibility, potential impact on health outcomes and cost effectiveness of practice-based link workers for people with multimorbidity living in deprived urban communities.

**Methods:**

A pragmatic exploratory randomised trial with wait-list usual care control and blinding at analysis was conducted during the COVID 19 pandemic (July 2020 to January 2021). Participants had two or more ongoing health conditions, attended a general practitioner (GP) serving a deprived urban community who felt they may benefit from a one-month practice-based social prescribing link worker intervention.. Feasibility measures were recruitment and retention of participants, practices and link workers, and completion of outcome data. Primary outcomes at one month were health-related quality of life (EQ-5D-5L) and mental health (HADS). Potential cost effectiveness from the health service perspective was evaluated using quality adjusted life years (QALYs), based on conversion of the EQ-5D-5L and ICECAP-A capability index to utility scoring.

**Results:**

From a target of 600, 251 patients were recruited across 13 general practices. Randomisation to intervention (*n* = 123) and control (*n* = 117) was after baseline data collection. Participant retention at one month was 80%. All practices and link workers (*n* = 10) were retained for the trial period. Data completion for primary outcomes was 75%. There were no significant differences identified using mixed effects regression analysis in EQ-5D-5L (MD 0.01, 95% CI -0.07 to 0.09) or HADS (MD 0.05, 95% CI -0.63 to 0.73), and no cost effectiveness advantages. A sensitivity analysis that considered link workers operating at full capacity in a non-pandemic setting, indicated the probability of effectiveness at the €45,000 ICER threshold value for Ireland was 0.787 using the ICECAP-A capability index.

**Conclusions:**

While the trial under-recruited participants mainly due to COVID-19 restrictions, it demonstrates that robust evaluations and cost utility analyses are possible. Further evaluations are required to establish cost effectiveness and should consider using the ICE-CAP-A wellbeing measure for cost utility analysis.

**Registration:**

This trial is registered on ISRCTN.

Title: Use of link workers to provide social prescribing and health and social care coordination for people with complex multimorbidity in socially deprived areas.

Trial ID: ISRCTN10287737.

Date registered 10/12/2019.

Link: https://www.isrctn.com/ISRCTN10287737

**Supplementary Information:**

The online version contains supplementary material available at 10.1186/s12875-024-02482-6.

## Introduction

Social prescribing is a means of enabling healthcare professionals (HCPs) to refer people to a range of local, non-clinical services to improve their health and well-being [1]. While the widespread use of the phrase for such referrals is relatively recent, social prescribing has been practiced for many decades by a variety of healthcare professionals, including general practitioners and occupational therapists [2]. More recently a holistic form of social prescribing has been described [3]. This form includes the use of a link worker, who is a non-health or social care professional who usually has training in coaching or behavioural change and an extensive knowledge of local community resources. They support people to identify their health and social needs and access community resources to improve health and wellbeing [4]. The link worker role has been identified as key to enabling successful social prescribing [5] and as such the holistic social prescribing model has been implemented in England [6]. Social prescribing has been proposed as a way to support self-management for people with long term conditions and address health inequalities [7], and is receiving increasing attention in Irish health policy and globally [8, 9].

Multimorbidity, the presence of two or more chronic conditions, is a significant challenge for patients and healthcare systems [10]. In deprived areas people develop multimorbidity earlier, are more likely to have complex combinations of physical and mental health conditions and have poorer quality of life than those living in affluent areas [11, 12].This results in higher consultation rates and healthcare costs [13, 14]. Evidence suggests that people with multimorbidity in areas of deprivation have reduced capacity for self-management due to psychosocial stressors, poorer mental health and lower perceived social support, potentially contributing to poorer outcomes and resulting health inequalities [15–20]. Social prescribing link workers could help to address the complex mix of psychosocial issues and multimorbidity in areas of deprivation.

However, evidence is limited with regard to potential impact and overall cost effectiveness, and uncertainty remains about how to implement social prescribing interventions and which populations benefit most, with considerable variation in practice [21, 22].We previously conducted a systematic review examining the effectiveness of link workers delivering social prescribing and only identified eight controlled studies, with five specifically targeting participants in areas of deprivation and two targeting participants with multimorbidity. We did not identify any cost effectiveness evaluations. Some studies, in particular those with controlled before after designs, struggled to recruit suitable controls. We concluded that there was very limited high-quality evidence to support the effectiveness of social prescribing [23].

Ireland’s Health Service Executive (HSE) is rolling out a Healthy Communities initiative that includes social prescribing in communities identified as experiencing risk factors for poorer health and wellbeing. Each community area with a population of approximately 20,000 would have a single link worker who would see 100 patients per annum with an intervention time tailored to the needs of the individual. Link worker location would vary depending on local circumstances, but some time embedded in a general practice is recommended [9]. GPs who self-identify as serving deprived areas in Ireland, who are members of Ireland’s Deep End network, are supportive of the embedded approach [24], and were keen to facilitate the evaluation of general practice-based link worker similar to the Glasgow ‘Deep End’ Links Worker Programme [25]. There is, however, a lack of evidence on the potential for social prescribing to reduce health inequalities [26, 27], and further evidence is needed to guide targeting of individuals most likely to benefit given the limited availability of link workers.

To address the ongoing evidence gap, we explored the feasibility and potential impact of primary care practice-based social prescribing link workers on health outcomes and costs for people with multimorbidity (the LinkMM intervention) living in deprived urban communities in Ireland.

## Methods

### Study design

We planned to conduct a definitive randomised controlled trial (RCT) and economic evaluation. However, the trial took place during the COVID-19 pandemic, which significantly affected recruitment and implementation of the intervention; therefore, we are now reporting the study as an exploratory RCT investigating feasibility, potential impact and cost effectiveness.

We conducted a pragmatic exploratory RCT to evaluate a general practice-based social prescribing link worker intervention for patients with multimorbidity in socially deprived areas compared to wait list controls receiving usual care. We also conducted a parallel cost utility analysis from the perspective of the public healthcare system following national guidance [28]. The trial protocol was informed by an uncontrolled pilot study in a single practice, which we published and is summarised below [29, 30]. The trial implementation was significantly affected by the COVID19 pandemic leading to adaptations to the original protocol to include measures of trial feasibility and to the intervention, which are described briefly in this paper. A separate parallel mixed methods process evaluation will explore implementation and acceptability of the intervention in detail. The protocol for this has been published elsewhere [31].

### Study settings

The exploratory RCT took place in primary care practices serving areas of deprivation in four cities within the Republic of Ireland, between July 2020 and January 2021 during varying levels of COVID-19 public health restrictions. These included a six-week level 5 lockdown when people were restricted to a 5km radius from their homes and most services and amenities were closed [29].

### Eligibility criteria

#### Participants

Participants were aged over 18, prescribed five or more medications, had two or more chronic conditions (multimorbidity), were attending a GP practice that self-identified as serving an area of deprivation (see under Practices below) and were identified by their GP as potentially benefitting from a link worker intervention. There were no predefined conditions, other than conditions should be chronic, lasting or expected to last more than six months.

Exclusion criteria included psychiatric/psychological morbidity or cognitive impairment that would impair capacity for informed consent, a terminal illness likely to lead to death or major disability during the study follow-up period, living in residential care or recently participating in a similar programme.

#### Practices

Practices in the Deep End Ireland group were invited to participate. Membership of this group is open to any practice that identifies as working in an area of deprivation. Currently there are approximately 40 practices on the Deep End mailing list. To be included in the study, practices had to serve at least two small areas defined as deprived by the Pobal HP deprivation index 2016 and be located in an urban area [32]. The Pobal HP deprivation index is Ireland’s most widely used social gradient metric and scores each small area (50 – 200 households) in terms of affluence or deprivation. The index uses information from Ireland’s census, such as employment, age profile and educational attainment, to calculate this score [32]. Practices also had to have a General Medical Scheme (GMS) list of > 1000 patients to ensure sufficient numbers of potentially eligible patients. The GMS scheme provides public medical care to approximately 40% of the Irish population and is a predominantly means-tested system of care. It provides eligible patients with free general practitioner visits, free hospital care and free medications (except for a prescription levy, currently €2.50 per item to a maximum of €25). Urban areas have more extreme concentration of disadvantage and affluence [33]. There are additional considerations in rural areas. Deprivation is more dispersed and harder to identify on an area level. Social prescribing programmes have to consider logistical challenges such as transport to community resources. This additional variation and adaptations required in rural areas in an already complex intervention was felt to be beyond the scope of the evaluation by the research team and so only urban practices were included.

#### Recruitment and randomisation

GPs in participating practices searched electronic health records (EHR) for all patients on five or more medications and selected all those they thought might benefit from the intervention. Five or more medications was used as a marker for multimorbidity to aid searching of electronic records for suitable participants. This was due to inconsistent coding of chronic conditions across practice EHRs. Medication counts have been shown to be a marker of multimorbidity [34]. GPs confirmed that potential participants had two or more chronic conditions. They also determined who they thought might benefit using their clinical judgement and knowledge of the patient’s circumstances. They were encouraged to consider patients with known social problems, addiction issues, mild mental health conditions, frequent attenders or those who did not attend health appointments. This pragmatic approach to patient identification was employed to replicate how patients would be referred to a practice-based link worker through their GP in a real-world setting. To minimise selection bias, GPs were asked to identify all potential participants and then used a random number sequence in Microsoft Excel to identify a random sample of potential participants, who were posted an information pack, consent form and baseline data collection form (Supplementary File 1). GPs followed up with a telephone call. Participating patients returned completed consent and baseline data collection forms by post to the research team. Participating patients could also request telephone assistance from the research team to complete baseline data collection. GPs were asked to continue recruitment on a rolling basis between July 2020 and December 2020 until they had met their target of 60 participants per practice.

Randomisation was carried out independently by the trial statistician (FB) using a computer-generated sequence after baseline data collection. Patients were stratified by practice and age (over or under 65 years). Allocation was blocked using random permuted blocks of sizes two and four. Due to the nature of the intervention, it was not possible to blind participants, link workers or GPs to the allocation. Blinding was implemented at the data analysis stage.

#### Intervention

The LinkMM intervention was based on an existing intervention, the Glasgow Deep End Links Worker Programme [25], and on an existing practice-based social prescribing service in Ireland. It targeted people living with multimorbidity in urban deprived communities and the link workers were based in GP practices. The short intervention period was determined in part by the available funding but was intended to reflect current resource allocation for social prescribing in Ireland where there is limited availability of link workers and also facilitated a wait-list control approach. We previously conducted a short uncontrolled pilot in a single practice to test acceptability of the intervention, feasibility of recruitment and, in consultation with stakeholders through an implementation advisory group, made refinements to the format of the initial assessment and selection of participants [30].

The majority of link workers were assigned to a single practice. Some link workers were assigned to a maximum of two practices, where the practices were small and in close proximity. Their intended workload was between two and three new referrals a week, or 60 participants over the course of the trial. After an initial hour-long assessment, where participants’ needs were identified, the link workers followed up over a one-month period with suggested community resources and support to access these. Initial assessments were intended to be in person, although follow-up contacts could be flexible. Link workers were also tasked with mapping local community resources and updating GPs on individual participant’s progress and resources identified. A detailed process evaluation including referrals to and use of community resources will be reported separately. We described the intervention in detail in the published protocol and further details are available in a TIDier checklist (Supplementary File 2) [29, 35].

#### Control

During the intervention period, the control group received usual care from their GP. Intervention and control participants were recruited on a rolling basis. After they had completed one-month follow-up data collection, the control group was invited to a one-off meeting with the link worker to identify their needs and received a list of suggested resources and activities.

#### Outcomes

Feasibility measures considered were recruitment and retention of participants, practices and link workers, and data completion for trial outcomes. Outcomes were based on the Core Outcome Set for Multimorbidity (COSMM) research, with health related quality of life (*(EQ-5D-5L) *[36] and mental health *(Hospital Anxiety and Depression Scale) *[37] as the primary trial outcomes [38]. Secondary outcomes included wellbeing, identified as a key measure in the HSE Social Prescribing Framework [9] and measured using the ICE-CAP A (*ICEpop CAPability measure for Adults*) [39], which can be converted to a utility score, providing an alternative way of assessing cost effectiveness. The ICE-CAP A is recommended for use by the National Institute for Health Care Excellence (NICE) alongside the EQ-5D-5L to capture the wider social benefits to the individual that are expected with this type of intervention [40] and the ICECAP-A has shown to be more appropriate to assess interventions aimed at improving wellbeing and mental health [41]. Other secondary outcome measures included treatment burden (*Multimorbidity Burden of Treatment Questionnaire*) [42], frequency of activity participation (*Frenchay Activity Index*) [43], and self-management behaviour (*Patient Activation Measure*) [44]. Healthcare utilisation measures assessed the direct impact of the intervention and facilitated costing for the economic analysis and included.

- Primary care attendances
- Out of hours primary care attendances
- Emergency Department attendances
- Hospital admissions (emergency) and length of stay
- Hospital outpatient visits.

#### Sample size

As an exploratory trial to determine feasibility a sample size is not required. The original protocol includes sample size calculations for a definitive RCT, which was 600 participants [29].

#### Data collection

We collected data at baseline and immediately after completion of one month of link worker support for the intervention group and at the same time point, prior to meeting the link worker, for the control group. Participants returned self-completed data collection forms either by post or over the phone with a member of the research team. A member of the research team extracted healthcare utilisation data from GP EHRs. Data on link worker activity (number of contacts and resources recommended) was collected from a bespoke client management database using Microsoft Access software.

#### Statistical analysis

The primary analysis was ‘intention-to-treat’ (ITT) including all randomised participants, all retained in the group to which they were allocated. For all patient reported outcomes, except patient activation, activity frequency and treatment burden (due to > 30% missing data), multiple imputation was used to impute missing data for 50 datasets under the assumption that data were missing at random. The imputation model included age and gender. We also conducted a complete case analysis for all variables. We used a mixed effects regression model controlling for baseline scores, age and gender and including general practice as a random effect to estimate mean differences (MD) between groups, 95% confidence intervals (CI) and p-values. We did a pre-planned per protocol evaluation of those who met with the link worker at least once and sub-group analyses based on gender, age and number of medications. Stata v15 was used for all data analysis [45].

#### Economic analysis

The economic analysis was a trial-based analysis with a time horizon of one month. Intervention costs were based on the costs of implementing the intervention, as recorded by the research team. The cost was allocated across all participants regardless of whether they met the link worker. Cost estimates for each resource activity were based on national guidance and data sources and, where necessary, adjusted to 2020 prices in Euros (€) using appropriate indices. Given the length of follow-up, neither costs nor outcomes were discounted (Supplementary Table 1 and 2a, Supplementary File 3).

#### Cost utility analysis

For the cost utility analysis, effectiveness was evaluated in terms of quality adjusted life years (QALYs), based on conversion of the EQ-5D-5L and ICECAP-A capability wellbeing index changes over a one month period to utility scoring as recommended by NICE [39, 46, 47]. Generalized linear model (GLM) regressions were used to estimate costs. Information on the marginal costs of the intervention was combined with effectiveness data to calculate incremental cost effectiveness ratios (ICERS).

Uncertainty in the analysis was addressed by estimating 95% CIs, hypothesis tests, and cost-effectiveness acceptability curves (CEACs), which link the probability of treatment being cost-effective to a range of potential threshold values (λ) that a health system may be willing to pay per additional QALY gained. In Ireland, threshold values in the range of €20,000 to €45,000 are generally recommended. The CEACs were estimated using a nonparametric bootstrapping technique, (*bootstrap* command, Stata 17), which jointly accounts for the correlation in the cost and effect data.

#### Sensitivity analyses

Sensitivity analyses considered link workers operating at full capacity, seeing 100 participants each per annum and reduced GP supports (reflecting reduced time required for recruitment in a non-research setting and reduced requirements for room hire due to more flexible working arrangements since the COVID-19 pandemic with link workers working remotely for non-patient facing work). An additional analysis incorporated higher wages for link workers, based on current HSE advertised salaries (Supplementary Table 2b and 2c, Supplementary File 3).

#### Public and patient involvement (PPI)

A multimorbidity patient advisory group supported PPI. They provided input on recruitment processes and data collection forms. The lead researcher (BK) presented the results to them and their comments informed the discussion. For further details, see GRIPP 2 form (Supplementary File 4).

## Results

### Feasibility outcomes

#### Practice and link worker recruitment and retention

There were 15 responses to the initial call to 40 practices for expressions of interest. Three practices were excluded: one was rural, one did not have compatible software to do the initial patient search and one did not have staff capacity to do the recruitment. Four co-located practices planned to share a link worker. Of the twelve initial practices, one dropped out, prior to patient recruitment due to staff illness and one reduced their recruitment commitment from 60 to 30 participants due to staff retirement. At this point the trial had commenced so four additional practices on the database were approached directly. Two of these had capacity to participate, one at full capacity and one at half capacity, sharing a link worker with the existing practice that reduced its commitment. This gave 13 of 14 recruited practices that were retained for the duration of the trial.

The proportion of registered patients in each practice living in a deprived or very deprived area as defined by the Pobal HP Deprivation Index varied from 28 to 88%. Variation was also evident in the proportion of patients on five or more medications (7% to 55%), and who were identified as potentially benefitting from the intervention (10% to 69%) (Supplementary Table 3, Supplementary File 5).

Ten link workers were successfully recruited and retained for the duration of the trial. Link workers were hired by the research team and had degree level qualifications in health promotion, psychology, social care and addiction supports. They all had experience providing one to one support to people with complex needs. They received a one-week induction covering social prescribing, motivational interviewing and behaviour change, had ongoing supervision from a member of the research team (an experienced community health care professional and manager) and formed a peer support network.

#### Intervention adaptations and delivery

In a deviation from planned intervention delivery, link workers were required to work remotely, conduct assessments virtually or meet those referred outdoors at various times during the trial depending on COVID 19 restrictions. There were level 5 COVID-19 public health restrictions (where people were restricted to within 5km of their home and indoor recreational services were closed) in place for 62% of intervention participants. This resulted in fewer contacts (16% vs 49% with 6 + contacts, *p* = 0.002) and fewer resources being recommended (7.8% vs 28.2% recommended 6 + resources, *p* = 0.03) than for participants where no restrictions were in place. Despite restrictions, link workers adapted using remote support, online resources and healthy lifestyle advice. Further details are available in the TIDieR checklist. (Supplementary File 2).

Of the 123 intervention participants, 102 (83%) met the link worker at least once, either face to face or via telephone. Those who did not meet the link worker were more likely to be on 10 + medications (80% vs 56%, *p* = 0.03) and had slightly higher anxiety scores (11.3 vs 9.3, *p* = 0.06) although this did not reach statistical significance (Supplementary Table 5, Supplementary File 5).

#### Participant recruitment, retention, and data completion

**Fig. 1:**
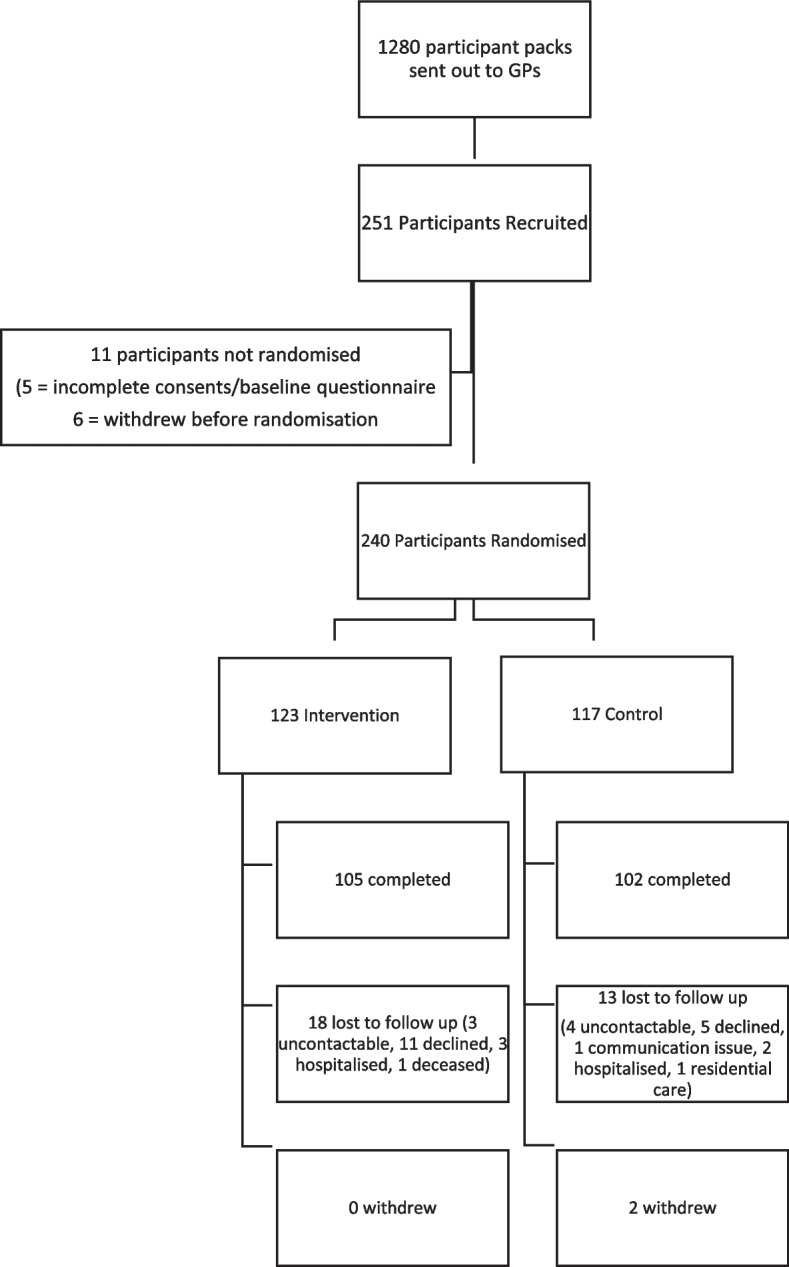
CONSORT flow diagram of participant recruitment and retention

There were approximately 2,103 eligible patients (Supplementary Table 3, Supplementary File 5). Based on a 50% recruitment rate, 1280 recruitment packs were sent to GP practices, who sent them to a randomly selected sample of eligible patients. We recruited 251 of the planned 600 participants, with a recruitment rate of 20%. Five participants had incomplete paperwork and 6 withdrew prior to randomisation, so 240 participants were randomised, of which 33 (14%) were lost to follow up (Fig. 1). There was a significant difference between those lost to follow up and other participants in education level with 42% vs 24% reporting primary education only respectively (*p* = 0.03) (Supplementary Table 4, Supplementary File 5).

Of the 240 participants, 180 had complete data for primary outcomes. Data completeness was higher for healthcare utilisation outcomes (96%) as a member of the research team collected this directly from practice EHRs. Completion for secondary outcomes was lower as more participants required telephone assistance to complete follow up data collection than expected. Given the number of outcomes this was onerous for participants and so the primary outcomes and ICECAP-A were prioritised. Patient activation (30%), treatment burden (45%) and the Frenchay Activity Index (FAI) (54%) had high rates of missing data at follow up as a result. Missing data at baseline was only notable for the FAI (30%).

#### Participant characteristics and outcome measures

Participants were 63% female, 59% were aged under 65, mean medication count was 12 (a marker of more complex multimorbidity), 12% were employed and 76% held a GMS card (a means tested entitlement to free healthcare indicating income in the lower third of the general population). Mean baseline anxiety scores were > 8 indicating possible caseness and treatment burden was moderate to high. Demographics were similar between groups (Table 1).

**Table 1:** Demographics and baseline scores of participants

	**Intervention** *n* = 123	**Control** *n* = 117
**Demographic characteristics**	n (%)	n (%)
Age Group		
18–24	2 (2)	0
25–44	15 (12)	9 (8)
45–64	53 (43)	61 (53)
65 +	53 (43)	45 (39)
Female	78 (65)	73 (64)
Medication count (Mean (SD))	11.7 (6.7)	12.3 (6.8)
GMS card holder	101 (87)	81 (75)
Primary education or below	35 (30)	29 (25)
Employed	19 (18)	10 (9)
Owner occupied housing	55 (50)	47 (44)
Living alone	32 (27)	36 (32)
Other language	9 (8)	4 (3)
Smoker	40 (34)	33 (30)
Alcohol 11 units +	19 (16)	14 (13)
**Patient reported outcomes**	**Mean (SD)**	**Mean (SD)**
EQ-5D-5l index	0.473 (0.4)	0.411 (0.4)
EQ-VAS	60.0 (20.3)	56.1 (21.2)
HADS	17.6 (8.9)	18.8 (8.9)
HADS Anxiety	9.6 (5.0)	10.5 (5.2)
HADS Depression	7.8 (4.7)	8.2(4.5)
ICECAP-A	0.723 (0 .2)	0.682 (0 .2)
PAM	53.7 (13.0)	53.5 (14.0)
MM Treatment Burden	20.0 (20.0)	22.1 (20.1)
Frenchay Activity Index	41.0 (8.9)	40.5 (9.1)

*GMS* General Medical Scheme: means tested scheme were card holders can avail of free GP visits, hospital care and medications. *EQ-5D-5L *a standardized measure of self-reported health-related quality of life that assesses 5 dimensions at 5 levels of severity where 1 is the preferred state of health, *EQ-VAS *a visual analogue scale of 0–100 with 0 indicating worst health and 100 best. *HADS-A *Hospital Anxiety and Depression Scale, Anxiety, where a score above 8 indicates possible caseness, *HADS-D *Hospital Anxiety and Depression Scale, Depression, where a score above 8 indicates possible caseness; ICECAP-A is a measure of capability and wellbeing with a range from 0 (worst wellbeing) to 1 (best wellbeing). Multimorbidity burden of treatment questionnaire categorises burden as no burden (score 0), low burden (score < 10), medium burden (10–22), high burden (> = 22); Patient Activation Measure assesses activation to manage health with scores from 0–100, 47 or lower indicating least activation, 67 or above highest; The Frenchay activity index categorises people as inactive, moderately active or very active based on work, leisure and domestic activity with scores from 15 (inactive) to 60 (very active)

In ITT analyses, there was no evidence of a difference between groups for primary or secondary outcomes (Table 2). An additional model, adjusting for multimorbidity severity using baseline medication count as an indicator, showed no effect on primary outcomes. Pre-planned sub-group analysis for age did not show any evidence of significant differences. There was a small increase in wellbeing (ICECAP-A) for men (MD: 0.07, 95% CI: 0.02 to 0.13, *p* = 0.01) in the gender sub-group analysis (Supplementary Table 6, Supplementary File 5). In the per protocol analysis there were no significant differences seen for any outcomes except the FAI, with those who met the link worker at least once reporting a higher score (MD:2.1, 95% CI:0.1–4.1, *p* = 0.04) (Supplementary Table 7, Supplementary File 5).

**Table 2:** Means and mean differences in patient-reported outcomes at one month

Outcome Measure	Intervention *n* = 123 Mean at follow up (SD)	Control *n* = 118 Mean at follow up (SD)	Adjusted^a^ Mean Difference ( 95% Confidence Interval)	*p* value
EQ-5D-5L	0.5 (0.4)	0.5 (0.4)	0.01 (-0.07 to 0.09)	0.81
EQ-VAS	60.9 (21.1)	57.9 (21.2)	1.60 (-3.00 to 6.20)	0.49
HADS^b^	16.8 (8.6)	17.9 (8.8)	0.05 (-0.63 to 0.73)	0.88
HADS-Anxiety	9.1 (4.8)	9.8 (5.1)	-0.18 (-0.99 to 0.64)	0.67
HADS- Depression	7.7 (4.6)	8.1 (4.5)	-0.03 (-0.84 to 0.79)	0.95
ICECAP-A	0.7 (0 .2)	0 .7 (0 .2)	0.03 (-0.01 to 0.06)	0.15
PAM^b^	58.1 (15.4)	55.8 (13.8)	2.49 (-1.13 to 6.11)	0.18
MM Treatment Burden^b^	18.5 (19.9)	20.7 (20.5)	-1.49 (-5.62 to 2.65)	0.48
Frenchay Activity Index^b^	40.5 (8.5)	40.3 (8.8)	1.24 (-0.85 to 3.33)	0.24

^a^Mixed effects regression model controlling for baseline scores, age and gender and including general practice as a random effect

^*^Complete case analysis as were not suitable for multiple imputation due to similarity to other scores for HADS and more than 30% missing data for other outcomes

*EQ-5D-5L *a standardized measure of self-reported health-related quality of life that assesses 5 dimensions at 5 levels of severity where 1 is the preferred state of health, *EQ-VAS *a visual analogue scale of 0–100 with 0 indicating worst health and 100 best. *HADS-A *Hospital Anxiety and Depression Scale, Anxiety, where a score above 8 indicates possible caseness, *HADS-D *Hospital Anxiety and Depression Scale, Depression, where a score above 8 indicates possible caseness, *ICECAP-A* is a measure of capability and wellbeing with a range from 0 (worst wellbeing) to 1 (best wellbeing). Multimorbidity burden of treatment questionnaire categorises burden as no burden (score 0), low burden (score < 10), medium burden (10–22), high burden (> = 22); Patient Activation Measure assesses activation to manage health with scores from 0–100, 47 or lower indicating least activation, 67 or above highest; The Frenchay activity index categorises people as inactive, moderately active or very active based on work, leisure and domestic activity with scores from 15 (inactive) to 60 (very active)

#### Health care utilisation

Intervention participants had a mean of 1.3 encounters with their GP in the month before baseline, including in person and telephone reviews, while control participants had 1.2. Both groups had a decrease in the mean GP and GP Nurse encounters in the follow up period, but the control group had a significantly greater decrease in GP visits (MD:0.35, 95% CI: 0.09 to 0.61, *p* = 0.008) and a smaller decrease in GP nurse visits (MD:-0.2, 95% CI (-0.30 to -0.06) (Table 3).

**Table 3:** Health Care Utilisation, total healthcare costs and quality adjusted life years

Health Care Utilisation						
	**Intervention**	**Control**	**Intervention**	**Control**		
**Outcome**	**Baseline** ***Mean (SD)***		**Follow Up** ***Mean (SD)***		**Mean Difference (95% Confidence Interval)**	***p *value**
GP Visits	1.3 (1.2)	1.2 (1.2)	1.1 (1.2)	0.7 (0.9)	0.35 (0.09 to 0.61)	0.008
GP Nurse Visits	0.3 (0.2)	0.3 (0.6)	0.1 (0.4)	0.3 (0.7)	-0.20 (-0.30 to -0.06)	0.004
OOH	0.03 (0.18)	0.07 (0.37)	0.05 (0.26)	0.07 (0.27)	-0.02 (-0.09 to 0.04)	0.51
A&E	0.03 (0.15)	0.07 (0.26)	0.04 (0.20)	0.08 (0.28)	-0.04 (-0.10 to 0.02)	0.20
LOHS (days)	0.14 (0.88)	0.24 (1.29)	0.01 (0.18)	0.12 (0.68)	-0.11 (-0.23 to 0.02)	0.09
OPD	0.33 (0.68)	0.32 (0.63)	0.28 (0.52)	0.19 (0.43)	0.08 (-0.03 to 0 .19)	0.15
**Healthcare costs Euro €**	267.35 (906.37)	379.91 (1315.41)	134.48 (245.48)	225.20 (735.34)		
**QALY** **(EQ-5D-5L)**			0.485 (0.372)	0.438 (0.363)	-0.004 (-0.042 to 0.034)	0.85
**QALY (ICECAP-A)**			0.720 (0.184)	0.693 (0.186)	0.015 (-0.005 to 0.035)	0.85

*GP *general practitioner, *PN *Practice Nurse, *OOH *Out of Hours, *A&E *Accident and Emergency, *LOHS *Length of Hospital Stay (emergency admissions)

Completeness of data:

Intervention: *Baseline –* 4% missing data for HCU, 8% EQ-5D-5L, 7% ICECAP-A

Control: *Baseline* – 2% missing data for HCU, 8% on EQ-5D-5L. 6% ICECAP-A

Intervention: *Follow-up –* 4% missing data for HCU, 18% for EQ-5D-5L, 17% for ICECAP-A

Control: *Follow-up* – < 1% missing data for HCU, 12% for EQ-5D-5L, 16% ICECAP-A

Health care utilisation: Mixed effects regression model controlling for baseline scores, age and gender and including general practice as a random effect

Cost analyses: GLM regression with log link function, Gamma variance function, estimated controlling for treatment group, baseline cost and general practitioner clustering. Where necessary unit costs were inflated using the health component of the consumer price index from the Central Statistics office [48]

QALYs Analyses: GLM regression model, with identity link function, Gaussian variance function, estimated controlling for treatment group, baseline EQ-5D-5L and general practitioner clustering

#### Economic analysis

**Fig. 2:**
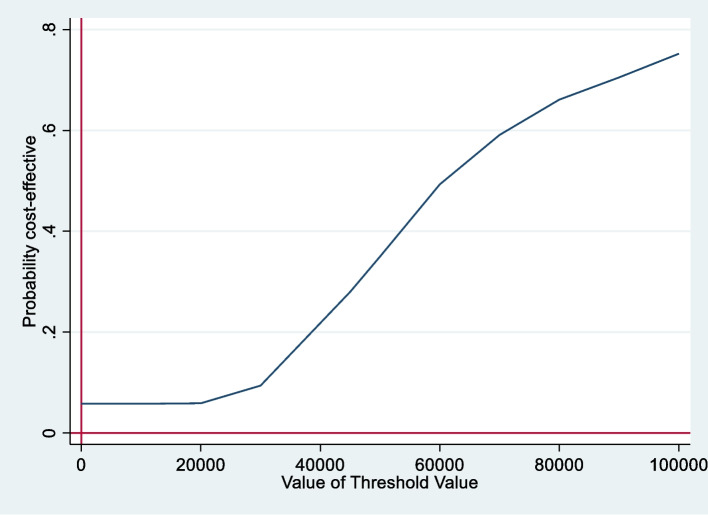
Cost effectiveness acceptability curve-ICECAP-A

The mean total cost was €1,191.48 for the intervention group and €225.20 for the control group. The intervention cost was €1.057 per intervention participant. The mean direct healthcare cost per patient was €134.48 for the intervention arm and €225.20 for the control arm (Table 3). The EQ-5D-5L showed a decrease in mean QALYs of -0.004 (-0.042, 0.034) [*p* = 0.852] for the intervention group at the one month follow up (Table 3). ICERs and cost-effectiveness probabilities were therefore not calculated for the EQ-5D-5L. The intervention was associated with an increase in mean QALYs of 0.015 (-0.005, 0.035) [*p* = 0.852] per patient at one month follow up, based on the ICECAP-A utility score. The expected cost-effectiveness results indicate that at alternative threshold values of €5,000, €10,000, €20,000, €30,000, and €45,000, the probability of the intervention being cost-effective was estimated to be 0.058, 0.058, 0.059, 0.09 and 0.280 respectively (Table 4 and Fig. 2).

**Table 4:** Incremental cost effectiveness analysis using ICECAP-A utility score

**Variable/ Analysis**			**Incremental Analysis** (Intervention minus Control)			
**Cost Analysis**						
**LinkMM Intervention Total Cost**			€1,057			
			**Intervention**		**Control**	
Mean Total Cost (SD)			€1,191.48 (245.48)		€225.20 (735.34)	
Difference in Mean Total Cost € (95% CI) [p-value]			€1,195.25 (856.90 to 1,533.59) [P < 0.001]			
**Health Outcome Analysis**						
			**Intervention**		**Control**	
Mean QALYS (SD)			0.720 (0.184)		0.693(0.186)	
Difference in Mean QALYs (95% CI) [p-value]			0.015 (-0.005 to 0.035) [*p* = 0.852]			
ICER (Difference in cost/difference in QALY)			€79,683			
***Probability (%) that the Intervention is Cost Effective for Threshold Value (λ)***						
λ = €0	λ = €5,000	λ = €10,000	λ = €20,000	λ = €30,000		λ = €45,000
0.058	0.058	0.058	0.059	0.094		0.280

In terms of sensitivity analysis, the full capacity model indicates that at a threshold value of €45,000 the probability of the intervention being cost effective is 0.787. Using higher HSE link worker salaries (as are being paid in the Healthy Communities initiative described in the introduction) reduces the probability of the intervention being cost effective to 0.553. (Supplementary Table 8a and 8b, Supplementary File 5).

## Discussion

This exploratory RCT of a social prescribing link worker intervention aimed to investigate the feasibility and potential impact and cost effectiveness of GP practice-based link workers providing social prescribing for patients with multimorbidity living in urban deprived communities. Feasibility of recruitment and retention of practices and link workers was demonstrated. However, there were significant challenges with patient recruitment and implementation as intended, due to the impact of the COVID-19 pandemic and issues relating to the target population around literacy and comfort with complex research consent procedures.

Eligibility criteria stated participants had to be attending a GP practice in a deprived area, rather than living in a deprived area, but recruited participants’ demographics indicated that we reached our target populationThose with lower educational attainment and more complex multimorbidity, indicated by medication counts, were less likely to complete follow up and engage with the link worker respectively, indicating the need for more intensive and targeted strategies to recruit and maintain adherence for this cohort of patients. We do not have data on the demographics of those who declined participation.

While there was no evidence of significant differences in patient outcomes detected in the primary analysis, pre-planned sub-group and per protocol analyses indicated an increase in wellbeing for male participants and the per protocol analysis indicated an increase in activity participation for those who saw the link worker at least once. One of the pre-planned sensitivity analyses exploring cost effectiveness using ICECAP-A-generated QALYs showed that if link workers had been operating at full capacity, there was a 79% probability of cost effectiveness at Irelands funding threshold of €45,000. Often there are more female participants in social prescribing programmes [23], but it is important for referrers to consider that men can also potentially benefit. Activity participation could be a mechanism by which social prescribing exerts an effect on other outcomes, although none was seen in this trial, possibly due to the short follow up period and lack of power. Although the sensitivity analysis suggests that further economic evaluations are worthwhile, the results should be interpreted with caution as it is assumed that the intervention would have a similar impact on wellbeing, despite the increased workload of the link workers.

### Comparison with existing literature

There are limited RCTs and no cost utility analysis of social prescribing link workers [23]. Our exploratory RCT targeted people with multimorbidity in areas of urban deprivation and aimed to address the evidence gap around which populations can benefit most from social prescribing [21]. Our demographics and baseline scores are comparable to studies that targeted similar populations [27, 49].

Our findings are consistent with other recent controlled trials of link worker interventions that reported lack of impact on health-related quality of life or mental health [27, 50, 51]. Interventions aiming to improve outcomes for people with multimorbidity have generally shown no difference in HRQoL [52]. Higher rates of primary care consultations have been found in other social prescribing studies [53, 54] possibly explained by the intervention identifying unmet need or a return to the primary care provider for support if ongoing connections with community resources have not been established after link worker support ends [5]. Our finding on increased frequency of activity participation for those who saw the link worker at least once I is consistent with an increase in self-reported exercise among those who saw a link worker three or more times reported in another study, but these findings could possibly indicate reverse causality [27].

Based on our systematic review, this is the first cost utility analysis of a social prescribing link worker intervention and addresses the gap in economic evaluations of social prescribing [23]. Existing economic evaluations focus on return on investment analysis or uncontrolled social return on investment analysis [55]. Our systematic review identified two controlled economic analyses: one return on investment analysis based on data from trials of the US IMPaCT intervention estimated a return of $2.47 for every $1 invested based on the intervention group having fewer and lower-cost hospital admissions; and one cost benefit analysis that found a reduction in ED costs for the intervention group, but this was offset by higher ambulatory care costs [53]. In England, the Ways to Wellness intervention was found to reduce hospital costs using a natural experiment. These reductions did not offset the interventions costs, but the evaluation did not consider other potential benefits [56]. A social return on investment (SROI) analysis of the British Red Cross social prescribing link worker intervention that aimed to reduce loneliness, reported an SROI of £3.42 per £1 spent [57]. These approaches have their limitations. The cost benefit approach is limited by a focus on healthcare costs, which may not decrease as expected, and does not consider wider benefits to society. Social return on investments consider potential societal benefits in detail, but there is no agreement on what benefits to count and how to assign value to them, making it hard to compare across programmes and interventions and there is a high risk of bias [58]. There is usually a lack of SROIs with a control comparison, meaning effect estimates tend to be exaggerated [59]. A cost utility analysis is the preferred method to establish costs effectiveness and allow comparison between interventions [60]. The findings from this research support the feasibility of conducting such analysis and indicates potential cost effectiveness under different circumstances from a capability well-being perspective suggesting further investigation is worthwhile.

### Strengths and limitations

Although the trial under recruited, it is the largest trial of social prescribing link workers with individual randomisation using a wait list control for this population to date and the only cost utility analysis, demonstrating feasibility of this approach. A process evaluation further exploring feasibility and implementation is underway [31]. Use of a relatively new measure, the ICECAP-A enabled a focus on wellbeing, as well as health related quality of life, and its incorporation in our cost utility analysis represents another way of assessing value [41, 61]. This approach may have merit given the potential impact of social prescribing on general wellbeing.

The major limitation of our trial in relation to patient health and economic outcomes was failure to recruit to target, because it was conducted during the COVID-19 pandemic when GPs were overwhelmed with work [62, 63] and likely had reduced capacity to recruit participants. Therefore the study is underpowered for PROMs and link workers were not operating at full capacity. Connection with community resources is a key process in social prescribing [5] and closure of community-based resources during pandemic restrictions also influenced implementation of the intervention.

There was no capacity to map individual patient addresses to provide an individual marker of deprivation, rather the focus was on GP practices that served areas of deprivation. There is also no objective demographic data on those who were invited, but declined, as we did not have their consent. While this information is important to better understand who is referred to link workers and who engages or does not, the burden of data collection had to be balanced with the realities of conducting research in general practice settings, which were further compounded by the impact of the COVID-19 pandemic on GP workload [64].

The wait-list control design and funding constraints limited the duration of the intervention and follow up, which was short at one month. While de facto social prescribing link worker interventions are often of a similar limited duration in service delivery settings [50, 65], longer interventions have been implemented for complex patients [27, 51]. The individual randomisation approach, while allowing for a suitable control, does create a risk of contamination of the control group who might be recommended resources by their GP after a GP interaction with the link worker. A randomised trial is the most rigorous form of evaluation to establish effectiveness, but it has limited ability to capture other benefits of social prescribing such as community engagement, that are better suited to other evaluation methods [66, 67].

There was no formal process of risk mitigation or adaptation of the intervention to comply with restrictions due to the unprecedented and unpredictable nature of the situation at the time. Rather than abandoning the trial, we thought the additional support at this time of crisis might be of benefit to people living with multimorbidity and we continued the intervention throughout the pandemic with online and outdoor meetings conducted depending on current public health guidelines at the time [68].

As well as the trial limitations discussed above (under recruitment, a short time-frame and the influence of the pandemic) the economic analysis has specific limitations. The EQ-5D-5L showed a slight decrease and so our analysis is based on ICECAP-A generated utility scores which is a new approach requiring further testing and replication [61]. Medication costs were not included, although prescriptions were not a direct intervention target and were unlikely to change significantly during the short intervention timeframe. Interventions involving social prescribing are likely to have longer term and wider societal consequences, such as return to employment, which were not captured in the economic analysis due to the healthcare provider perspective.

### Implications

Recruitment in this target group is challenging even under ideal circumstances. Additional supports for in-person completion of data collection with fewer outcomes could enhance recruitment and follow-up data collection in future studies and mitigate against the exclusion of vulnerable participants. Recruiting patients during scheduled chronic disease management reviews is an approach that has been successful in facilitating this in other studies [51]. There is still no core outcome set for social prescribing interventions although work is underway to develop this. Such an outcome set, with some simple measures of intermediate outcomes like activity participation alongside health and wellbeing measures could help to reduce the need for multiple outcomes measures.

Based on our findings and those of other studies [27, 51], a one-month intervention is unlikely to be long enough to properly assess effectiveness of social prescribing interventions for more complex patient populations such as those living with multimorbidity, particularly in more deprived communities. A flexible and more personalised approach to intervention duration, which was supported by our PPI group, would allow further tailoring of the intervention.

Contamination of the control group who may be recommended community resources directly by their GPs, because of GP interaction with the link worker, could be limited by a cluster approach. This would also allow for flexibility in the duration of the intervention. Previous cluster type designs have struggled to get suitable controls [27, 50], but a stepped wedge design, that involved practice recruitment as a regional or national service was being implemented, would allow a wait-list type approach for practices, could help to replicate GPs usual selection processes and minimise control group differences.

While there is uncertainty around the cost effectiveness of social prescribing link workers, our analysis provides important information on potential cost effectiveness under different scenarios. If ongoing roll out of link workers is going to continue there should be close attention to the costs and a robust cost effectiveness analysis should always be conducted.

## Conclusions

Although failing to recruit to target due to COVID-19 this exploratory trial adds to the much-needed evidence to inform the implementation and evaluation of social prescribing link worker interventions. A cost sensitivity analysis suggests that under ideal circumstances with link workers working at capacity, there is a 78% probability of cost effectiveness, but further robust evaluations are needed prior to any recommendations or decisions about widespread implementation. What we can say for now is that studies like this are feasible, even in difficult circumstances, that the duration of the trial is important for impact and that outcomes need to incorporate health and wellbeing dimensions.

### Supplementary Information

Supplementary Material 1.

Supplementary Material 2.

Supplementary Material 3.

Supplementary Material 4.

Supplementary Material 5.

## Data Availability

Data will be stored for seven years in line with RCSI data management policy and shared at the time of publication where facilities permit and under ethical and data protection requirements. Once final data analysis has been undertaken and peer reviewed publications secured, anonymised data arising from this study may be accessed by contacting the PI and data may be placed on publicly accessible sites such as the Irish Social Science Data Archive (ISSDA). Researchers who wish to access the data can submit a request to the ISSDA and can use the data for research or teaching purposes with appropriate attribution and citation.
