# Supplementary material for: An exploratory randomised trial investigating feasibility, potential impact and cost effectiveness of link workers for people living with multimorbidity attending general practices in deprived urban communities
Source: BMC Prim Care. 2024 Jun 28;25:233. doi: 10.1186/s12875-024-02482-6 (PMC11212363; doi:10.1186/s12875-024-02482-6)
Supplement: Supplementary file 1 — Supplementary Material 1. [file 12875_2024_2482_MOESM1_ESM.docx]

| LinkMM: Use of link workers to provide social prescribing and health and social care coordination for people with complex multimorbidity in socially deprived areas. |
| --- |

## LinkMM Study Demographic, socioeconomic and health baseline questionnaire

We are interested in finding out a bit about you and your health. Answering this questionnaire will provide us with information that we can use to see if meeting with a link worker can help improve peoples’ health over time. The questionnaire isn’t very long and should take about 20-30 minutes to complete. The questionnaire is in two sections as follows:

| Section A | Questionnaires about your health and well being |
| --- | --- |
| Section B | Some details about you |

## Instructions for completing the questionnaire

- Please answer as fully as you can.
- For questions with multiple choice options, please tick the option that best describes you.
- A reply-paid envelope is provided for you to return the questionnaire to the research team.
- If you have any questions or would like to fill this form out over the phone or in person please contact me, Bridget Kiely, on 01 4774018.

## CONFIDENTIALITY

Only the researchers involved in this study will see your answers and they will ensure complete confidentiality.

## THANK YOU

We appreciate you taking the time to complete this questionnaire and participate in this study. Your response is very important and will provide us with valuable information to help us find out if link workers improve health for people with multiple medical conditions

## Contact details

Dr Bridget Kiely, PhD student, 014774018, bridgetkiely@rcsi.com

| **Section A** | **Questionnaires about your health and well being** | |
| --- | --- | --- |
| **Under each heading, please tick the ONE box that best describes your health TODAY.** | | |
| MOBILITY | |  |
| I have no problems in walking about | | ❑ |
| I have slight problems in walking about | | ❑ |
| I have moderate problems in walking about | | ❑ |
| I have severe problems in walking about | | ❑ |
| I am unable to walk about | | ❑ |
| SELF-CARE | |  |
| I have no problems washing or dressing myself | | ❑ |
| I have slight problems washing or dressing myself | | ❑ |
| I have moderate problems washing or dressing myself | | ❑ |
| I have severe problems washing or dressing myself | | ❑ |
| I am unable to wash or dress myself | | ❑ |
| USUAL ACTIVITIES *(e.g. work, study, housework, family or leisure activities)* | |  |
| I have no problems doing my usual activities | | ❑ |
| I have slight problems doing my usual activities | | ❑ |
| I have moderate problems doing my usual activities | | ❑ |
| I have severe problems doing my usual activities | | ❑ |
| I am unable to do my usual activities | | ❑ |
| PAIN / DISCOMFORT | |  |
| I have no pain or discomfort | | ❑ |
| I have slight pain or discomfort | | ❑ |
| I have moderate pain or discomfort | | ❑ |
| I have severe pain or discomfort | | ❑ |
| I have extreme pain or discomfort | | ❑ |
| ANXIETY / DEPRESSION | |  |
| I am not anxious or depressed | | ❑ |
| I am slightly anxious or depressed | | ❑ |
| I am moderately anxious or depressed | | ❑ |
| I am severely anxious or depressed | | ❑ |
| I am extremely anxious or depressed | | ❑ |

| We would like to know how good or bad your health is TODAY. |
| --- |
| This scale is numbered from 0 to 100. |
| 100 means the best health you can imagine. 0 means the worst health you can imagine. |
| Mark an X on the scale to indicate how your health is TODAY. |
| Now, please write the number you marked on the scale in the box below. |

YOUR HEALTH TODAY =

10

0

20

30

40

50

60

80

70

90

100

5

15

25

35

45

55

75

65

85

95

The worst health you can imagine

**ABOUT YOUR OVERALL QUALITY OF LIFE**

Please indicate which statements best describe your overall quality of life at the

moment by placing a tick () in **ONE** box for each of the five groups below.

**1. Feeling settled and secure**

|  |
| --- |
|  |
|  |
|  |

I am able to feel settled and secure in **all** areas of my life 4

I am able to feel settled and secure in **many** areas of my life 3

I am able to feel settled and secure in **a few** areas of my life 2

I am **unable** to feel settled and secure in **any** areas of my life 1

**2. Love, friendship and support**

|  |
| --- |
|  |
|  |
|  |

I can have **a lot** of love, friendship and support 4

I can have **quite a lot** of love, friendship and support 3

I can have **a little** love, friendship and support 2

I **cannot** have **any** love, friendship and support 1

**3. Being independent**

|  |
| --- |
|  |
|  |
|  |

I am able to be **completely** independent 4

I am able to be independent in **many** things 3

I am able to be independent in **a few** things 2

I am **unable** to be at all independent 1

**4. Achievement and progress**

|  |
| --- |
|  |
|  |
|  |

I can achieve and progress in **all** aspects of my life 4

I can achieve and progress in **many** aspects of my life 3

I can achieve and progress in **a few** aspects of my life 2

I **cannot** achieve and progress in **any** aspects of my life 1

**5. Enjoyment and pleasure**

|  |
| --- |
|  |
|  |
|  |

I can have **a lot** of enjoyment and pleasure 4

I can have **quite a lot** of enjoyment and pleasure 3

I can have **a little** enjoyment and pleasure 2

I **cannot** have **any** enjoyment and pleasure 1

Please ensure you have only ticked **ONE** box for each of the five groups.

**Tick the box beside the reply that is closest to how you have been feeling in the past week.**

**Don’t take too long over you replies: your immediate is best.**

| **I feel tense or 'wound up':** | **I feel as if I am slowed down:** |
| --- | --- |
| Most of the time □ | Nearly all the time □ |
| A lot of the time □ | Very often □ |
| From time to time, occasionally □ | Sometimes □ |
| Not at all □ | Not at all □ |
|  |  |
| **I still enjoy the things I used to enjoy:** | **I get a sort of frightened feeling like 'butterflies' in the stomach:** |
| Definitely as much □ | Not at all □ |
| Not quite so much □ | Occasionally □ |
| Only a little □ | Quite Often □ |
| Hardly at all □ | Very Often □ |
|  |  |
| **I get a sort of frightened feeling as if something awful is about to happen:** | **I have lost interest in my appearance:** |
| Very definitely and quite badly □ | Definitely □ |
| Yes, but not too badly □ | I don't take as much care as I should □ |
| A little, but it doesn't worry me □ | I may not take quite as much care □ |
| Not at all □ | I take just as much care as ever □ |
|  |  |
| **I can laugh and see the funny side of things:** | **I feel restless as I have to be on the move:** |
| As much as I always could □ | Very much indeed □ |
| Not quite so much now □ | Quite a lot □ |
| Definitely not so much now □ | Not very much □ |
| Not at all □ | Not at all □ |
|  |  |
| **Worrying thoughts go through my mind:** | **I look forward with enjoyment to things:** |
| A great deal of the time □ | As much as I ever did □ |
| A lot of the time □ | Rather less than I used to □ |
| From time to time, but not too often □ | Definitely less than I used to □ |
| Only occasionally □ | Hardly at all □ |
|  |  |
| **I feel cheerful:** | **I get sudden feelings of panic:** |
| Not at all □ | Very often indeed □ |
| Not often □ | Quite often □ |
| Sometimes □ | Not very often □ |
| Most of the time □ | Not at all □ |
|  |  |
| **I can sit at ease and feel relaxed:** | **I can enjoy a good book or radio or TV program:** |
| Definitely □ | Often □ |
| Usually □ | Sometimes □ |
| Not Often □ | Not often □ |
| Not at all □ | Very seldom □ |

Please check you have answered all the questions

**Below are some statements that people sometimes make when they talk about their health. Please indicate how much you agree or disagree with each statement as it applies to you personally by circling your answer. There are no right or wrong answers, just what is true for you. If the statement does not apply to you, circle N/A.**

| **1. I am the person who is responsible for taking care of my health.** | Disagree Strongly | Disagree | Agree | Agree Strongly | N/A |
| --- | --- | --- | --- | --- | --- |
| **2. Taking an active role in my own health care is the most important thing that affects my health.** | Disagree Strongly | Disagree | Agree | Agree Strongly | N/A |
| **3. I am confident I can help prevent or reduce problems associated with my health.** | Disagree Strongly | Disagree | Agree | Agree Strongly | N/A |
| **4. I know what each of my prescribed medications do.** | Disagree Strongly | Disagree | Agree | Agree Strongly | N/A |
| **5. I am confident that I can tell whether I need to go to the doctor or whether I can take care of a health problem myself.** | Disagree Strongly | Disagree | Agree | Agree Strongly | N/A |
| **6. I am confident that I can tell a doctor or nurse concerns I have even when he or she does not ask.** | Disagree Strongly | Disagree | Agree | Agree Strongly | N/A |
| **7. I am confident that I can carry out medical treatments I may need to do at home.** | Disagree Strongly | Disagree | Agree | Agree Strongly | N/A |
| **8. I understand my health problems and what causes them** | Disagree Strongly | Disagree | Agree | Agree Strongly | N/A |
| **9. I know what treatments are available for my health problems.** | Disagree Strongly | Disagree | Agree | Agree Strongly | N/A |
| **10. I have been able to maintain lifestyle changes, like healthy eating or exercising.** | Disagree Strongly | Disagree | Agree | Agree Strongly | N/A |
| **11. I know how to prevent problems with my health.** | Disagree Strongly | Disagree | Agree | Agree Strongly | N/A |
| **12. I am confident I can work out solutions when new problems arise with my health.** | Disagree Strongly | Disagree | Agree | Agree Strongly | N/A |
| **13. I am confident that I can maintain lifestyle changes, like healthy eating and exercising, even during times of stress.** | Disagree Strongly | Disagree | Agree | Agree Strongly | N/A |

**We are interested in finding out about the effort you have to make to look after your health and how this impacts on your day-to-day life.**

**Please tell us how much difficulty you have with the following:** (Please tick the box that most applies to you)

|  | Extremely Difficult | Very Difficult | Quite Difficult | A little Difficult | Not Difficult | Does not apply |
| --- | --- | --- | --- | --- | --- | --- |
| 1. Taking lots of medications | 5 | 4 | 3 | 2 | 1 | 0 |
| 1. Remembering how and when to take medication | 5 | 4 | 3 | 2 | 1 | 0 |
| 1. Paying for prescriptions, over the counter medication or equipment | 5 | 4 | 3 | 2 | 1 | 0 |
| 1. Collecting prescription medication | 5 | 4 | 3 | 2 | 1 | 0 |
| 1. Monitoring your medical conditions (e.g. checking your blood pressure or blood sugar, monitoring your symptoms etc.) | 5 | 4 | 3 | 2 | 1 | 0 |
| 1. Arranging appointments with health professionals | 5 | 4 | 3 | 2 | 1 | 0 |
| 1. Seeing lots of different health professionals | 5 | 4 | 3 | 2 | 1 | 0 |
| 1. Attending appointments with health professionals (e.g. getting time off work, arranging transport etc.) | 5 | 4 | 3 | 2 | 1 | 0 |
| 1. Getting health care in the evenings and at weekends | 5 | 4 | 3 | 2 | 1 | 0 |
| 1. Getting help from community services (e.g. physiotherapy, district nurses etc.) | 5 | 4 | 3 | 2 | 1 | 0 |
| 1. Obtaining clear and up-to-date information about your condition | 5 | 4 | 3 | 2 | 1 | 0 |
| 1. Making recommended lifestyle changes (e.g. diet and exercise etc.) | 5 | 4 | 3 | 2 | 1 | 0 |
| 1. Having to rely on help from family and friends | 5 | 4 | 3 | 2 | 1 | 0 |

**The table below is about your usual activities. Please circle the option that fits best.**

| **In the last 3 months how often have you undertaken: *(please circle)*** | | | | |
| --- | --- | --- | --- | --- |
| **1. Preparing main meals** | Never | Less than once a week | 1-2 times per week | Most days |
| **2. Washing up after meals** | Never | Less than once a week | 1-2 times per week | Most days |
| **3. Washing clothes** | Never | 1-2 times in 3 months | 3-12 times in 6 months | At least weekly |
| **4. Light housework** | Never | 1-2 times in 3 months | 3-12 times in 6 months | At least weekly |
| **5. Heavy housework** | Never | 1-2 times in 3 months | 3-12 times in 6 months | At least weekly |
| **6. Local Shopping** | Never | 1-2 times in 3 months | 3-12 times in 6 months | At least weekly |
| **7. Social occasions** | Never | 1-2 times in 3 months | 3-12 times in 6 months | At least weekly |
| **8 Walking outside for > 15 minutes** | Never | 1-2 times in 3 months | 3-12 times in 6 months | At least weekly |
| **9. Actively pursuing hobby** | Never | 1-2 times in 3 months | 3-12 times in 6 months | At least weekly |
| **10. Driving car/going on bus** | Never | 1-2 times in 3 months | 3-12 times in 6 months | At least weekly |
| **In the last 6 months how often have you undertaken:** | | | | |
| **11. Travel outing/car ride** | Never | 1-2 times in 6 months | 3-12 times in 6 months | At least weekly |
| **12. Gardening** | Never | Light | Moderate | Heavy/All necessary |
| **13. Household maintenance** | Never | Light | Moderate | Heavy/All necessary |
| **14. Reading books** | None | 1 in 6 months | Less than 1 in 2 weeks | More than 1 every 2 weeks |
| **15. Gainful work** | None | Up to 10 hours/week | 10-30 hours/week | Over 30 hours/week |

| **Section B** | **Your demographic details** |
| --- | --- |
| **Age group *(please tick)*** | |
| 18-24 | □ |
| 25-44 | □ |
| 45-64 | □ |
| 65+ | □ |
| **Gender *(please tick)*** | |
| Female □  Male □ | |
| **Educational attainment *(please tick)*** | |
| Primary or below | □ |
| Lower secondary | □ |
| Higher secondary | □ |
| Post leaving certificate | □ |
| Third level | □ |
| **Employment status *(please tick)*** | |
| Employed | □ |
| Looking for first job | □ |
| Unemployed | □ |
| Unable to work due to long term illness or disability | □ |
| Looking after home/family | □ |
| Retired | □ |
| Student | □ |
| **If you are/were employed what do you/did you do for work *(please write)*** | |
|  | |
| **Housing status *(please tick)*** | |
| Owner occupied | □ |
| Private rented | □ |
| Local authority | □ |
| Other | □ |
| **Family unit *(please tick)*** | |
| Live alone | □ |
| Live with family and/or partner/spouse | □ |
| Live with friends | □ |
| Live with flat or housemates | □ |
| Other | □ |
| **Do you speak a language other than English or Irish at home? *(please tick)*** | |
| Yes □ | No □ |
| **How many cigarettes or roll ups would you smoke a day? *(please tick)*** | |
| 0 | □ |
| 1-5 | □ |
| 6-10 | □ |
| 10-15 | □ |
| 15+ | □ |
| **How often do you drink alcohol? *(please tick)*** | |
| Never/not in last year | □ |
| < monthly | □ |
| 1-3 times per month | □ |
| Once a week | □ |
| 2-3 times a week | □ |
| 4+ time per week | □ |
| **How many alcohol units would you usually have at a time? *(please tick)***  *A pint/can of beer=2 units, A glass of wine=1.5 units, One standard measure of spirits= 1 unit, One alcopop= 1.5 units. So 4 pints is 8 units, half a bottle of wine is 5 units.* | |
| 1-2 | □ |
| 3-4 | □ |
| 5-6 | □ |
| 7-8 | □ |
| 9+ | □ |

**Thank you** for completing this questionnaire. Please return it via post via the stamped addressed envelope enclosed. If you have any queries about this study or the questionnaire please contact

Dr Bridget Kiely, PhD student, 014774018, bridgetkiely@rcsi.com
