# Supplementary material for: An exploratory randomised trial investigating feasibility, potential impact and cost effectiveness of link workers for people living with multimorbidity attending general practices in deprived urban communities
Source: BMC Prim Care. 2024 Jun 28;25:233. doi: 10.1186/s12875-024-02482-6 (PMC11212363; doi:10.1186/s12875-024-02482-6)
Supplement: Supplementary file 2 — Supplementary Material 2. [file 12875_2024_2482_MOESM2_ESM.docx]

Supplementary File 2. TIDieR Checklist for Intervention Description

|  | **The TIDieR (Template for Intervention Description and Replication) Checklist** |
| --- | --- |
|  |  |
|  | **BRIEF NAME** |
| **1.** | LinkMM: Social prescribing link workers for people with multimorbidity |
|  | **WHY** |
| **2.** | People with multimorbidity experience worse health outcomes and quality of life despite higher health care utilisation than those without. Psychosocial stressors, poorer mental health, reduced self -efficacy and fragmented healthcare systems are all potential contributors to this. There are limited evidence based interventions in primary care known to improve outcomes for people with multimorbidity. There is some evidence that social prescribing link workers can help address these issues, improving patient experience of care and reducing hospitalisations when delivered alongside chronic disease programmes. |
|  | **WHAT** |
| **3.** | Resources: A social prescribing link worker (a non-health or social care professional skilled in behaviour change and with an in depth knowledge of local community resources) based in primary care practices. |
| **4.** | Procedures:  GPs received training on the trial procedures and the link worker role  Link workers were hired by the research team and received a one-week induction.  GPs recruited patients with two or more chronic conditions that they believed would benefit from a link worker intervention.  The core components of the intervention were an initial assessment, goal setting, referral to community resources, follow up support and a final assessment to review progress.  The initial face-to-face or telephone assessment with the link worker to identified patients individual needs and helped them set personal goals. This usually took an hour, but may have taken longer or be spread over several encounters depending on the individuals requirements.  The link worker then followed up, either in person, by phone or text message with the patient to recommend community resources and provide support in connecting with these or achieving personal goals. Community resources participants were referred to included chronic disease self-management programmes, on line mental health resources and exercise classes. The link worker also liaised with relevant healthcare providers if appropriate and with patients consent.  At the end of the month, the link worker and patient reviewed progress and the link worker provided a summary report to the GP practice.  Link workers also mapped local community resources and updated GP staff on suitable resources either through formal or informal meetings.  Common resources recommended to participants by link workers included online chronic disease management programmes, online mental health supports, personal hobbies and healthy lifestyle advice. |
|  | **WHO PROVIDED** |
| **5.** | Social prescribing link workers are non-health or social care professionals skilled in behaviour change and with an in depth knowledge of local community resources. They typically have a background qualification in the areas of health or social care, with skills in behaviour change such as coaching or motivational interviewing. They are experienced in supporting people with complex needs one to one and in working in community and/or healthcare settings.  Link workers were hired by the research team and had degree level qualifications in health promotion, psychology, social care and addiction supports. They all had experience providing one to one support to people with complex needs. They received a one week induction covering social prescribing, motivational interviewing and behaviour change, |
|  | **HOW** |
| **6.** | Social prescribing link workers met individual patients face to face in the practice for initial assessment, although this was adapted to suit individual patient needs and public health restrictions, such as home visits, outdoor walks or telephone calls. Follow up contacts were either face to face, by telephone, WhatsApp or SMS messages or other means preferred by patient. Information on resources was shared verbally, by SMS/WhatsApp, written or by email. |
|  | **WHERE** |
| **7.** | The intervention primarily took place in the GP practice or remotely by telephone, but in some cases involved meetings outside the practice such as accompanying a patient to a community resource or activity. |
|  | **WHEN and HOW MUCH** |
| **8.** | Social prescribing link workers aimed to meet all participants at least once, with the number of follow ups tailored to the needs of the individual. The median number of follow-ups was 3 (range 1-20) and 85% were by telephone. During these follow-ups link workers provided a variety of support: informational (62%), instrumental (3%), appraisal (15%) and emotional (21%). |
|  | **TAILORING** |
| **9.** | The activities and resources recommended to patients was based upon their individual needs and preferences. The mode of contact, amount and type of support (informational, instrumental, emotional or appraisal) was tailored to the individual patients needs and preferences. |
|  | **MODIFICATIONS** |
| **10.** | Pandemic related restrictions lead to modifications such as limited face to face contact in order to comply with public health recommendations. There was also a focus on resources that were available on-line or on individual physical and well-being during lock downs.  Link workers had to work remotely and this affected communications with the GPs about resources, especially if no formal arrangements to meet were in place. Some practices held on line meetings with the link worker in place of formal in person meetings. |
|  | **HOW WELL** |
| **11.** | Data on fidelity was collected from the Access client management database used by the link workers for client records. 120/123 intervention participants were available to meet the link worker. Two participants withdrew after randomisation and one died. 92 participants had a record of a first assessment with the link worker. |
| **12.^ǂ^** | Overall 80% of the components of the intervention were implemented. 87% completed a first assessment, 79% set goals, 85% were recommended community resources, 85% completed a final assessment. However this did not translate into participants achieving their goals with only 51% stating Yes for goal achievement rating, possibly due to many resources being closed during COVID-19 restrictions. |
