# Supplementary material for: An exploratory randomised trial investigating feasibility, potential impact and cost effectiveness of link workers for people living with multimorbidity attending general practices in deprived urban communities
Source: BMC Prim Care. 2024 Jun 28;25:233. doi: 10.1186/s12875-024-02482-6 (PMC11212363; doi:10.1186/s12875-024-02482-6)
Supplement: Supplementary file 3 — Supplementary Material 3. [file 12875_2024_2482_MOESM3_ESM.docx]

| Supplementary Table 1. Healthcare costs used for analysis | | | |
| --- | --- | --- | --- |
| **Healthcare Resources** | **Activity** | **Unit Cost €** | **Source** |
| GP Visits: | Per Visit | 52 | Smith S, Jiang J, Normand C and O’Neill C. Unit costs for non-acute care in Ireland 2016—2019 [version 1; peer review: 2 approved]. HRB Open Res 2021, 4:39 (https://doi.org/10.12688/hrbopenres.13256.1) |
| PN Visits | Per Visit | 41 | Gillespie P, Carter L, McIntosh C, Gethin G. Estimating the health-care costs of wound care in Ireland. Journal of Wound Care. 2019;28(6):324-30.21 |
| OOH Visits | Per Visit | 41.63 | HSE GP contract accessed from <https://www.imo.ie/i-am-a/gp/gms-contract-agreements-a/> 14/09/2022 |
| A&E Visits | Per Visit | 289 | HPO |
| Outpatient Visits | Per Visit | 140 | HPO |
| Inpatient Days | Per Day | 747 | HPO |
| Inpatient Nights | Per Night | 962 | HPO |
| GP general practitioner, (PN) Practice Nurse, A&E accident and emergency (HPO) Healthcare Pricing Office Admitted Price List accessed from https://www.hpo.ie/abf/ABF2022AdmittedPatientPriceList.pdf. Where necessary unit costs were inflated using the health component of the consumer price index from the Central Statistics Office as per national guidelines. | | | |

Supplementary File 3. Additional economic data: health care costs and costs for intervention and sensitivity models

| Supplementary Table 2a. Intervention costs as implemented in trial | **N= 238** |
| --- | --- |
| **Category** | **Euro** |
|  | € |
| Link worker salaries (10 at RCSI Research Assistant, Level 1, Point 17 for 6 months) | 188,251 |
| Training (including room hire, time of link workers and trainers) | 25,011 |
| Supervision (1 hr per month supervision from PM (RCSI Research Fellow level 3, Point 1) | 1,609 |
| GP practice hosting (cover room hire, admin costs and GP time) | 35,100 |
| Phones (smart phone and €15 per month top up) | 1,700 |
|  |  |
| **Total** | **251,671** |
|  |  |
| **Average cost per participant** | 1,057 |

| Supplementary Table 2b. Interventions costs for full capacity sensitivity analysis | **N= 500*** |
| --- | --- |
| **Category** | **Euro** |
|  | € |
| Link worker salaries (10 at RCSI Research Assistant, Level 1, Point 17) | 188,251 |
| Training (including room hire, time of link workers and trainers) | 25,011 |
| Supervision (1 hr per month supervision from PM (RCSI Research Fellow level 3, Point 1) | 1,609 |
| GP practice hosting† (cover admin costs and GP time 1 session per month at 210) | 15,100 |
| Phones (smart phone and €15 per month top up) | 1,700 |
|  |  |
| **Total** | 231,671 |
|  |  |
| Average cost per participant seen | 463 |
| *Based on HSE Social Prescribing Framework workload where each link worker would see 100 patients per annum, or 50 in six months, so ten link workers would see 500 patients in six months.  † Room hire excluded as this is not usually offered in HSE hosting arrangements as the link worker would be based within HSE or host organisation premises for some of their time. | |

| Supplementary Table 2c. Intervention costings for full capacity sensitivity analysis with higher HSE salaries* | **N= 500** |
| --- | --- |
| **Category** | **Euro** |
|  | € |
| Link worker salaries (10 at HSE Grade V mid-point) | 306,149 |
| Training (including room hire, time of link workers and trainers) | 40,287 |
| Supervision (HSE Grade VII mid-point, 1 hr per month supervision) | 6,334 |
| GP practice hosting (cover admin costs and GP time 1 session per month at 250) | 17,500 |
| Phones (smart phone and €15 per month top up) | 1,700 |
|  |  |
| **Total** | **369,471** |
|  |  |
| Average cost per participant seen | 739 |
| * Health Service Executive roles are being advertised at a higher rate than was paid in the trial. GP sessions costed at current HSE rates. | |
