# Supplementary material for: An exploratory randomised trial investigating feasibility, potential impact and cost effectiveness of link workers for people living with multimorbidity attending general practices in deprived urban communities
Source: BMC Prim Care. 2024 Jun 28;25:233. doi: 10.1186/s12875-024-02482-6 (PMC11212363; doi:10.1186/s12875-024-02482-6)
Supplement: Supplementary file 4 — Supplementary Material 4. [file 12875_2024_2482_MOESM4_ESM.docx]

Guidance for reporting involvement of patients and public (GRIPP) 2.

| **1: Aim** |
| --- |
| The aim of the PPI was to provide the perspective of people living with multimorbidity on specific issues relating to recruitment identified during the pilot study. |
| **2: Methods** |
| An advisory panel of twelve people living with multimorbidity was recruited via existing networks of students on a PhD program in multimorbidity. The panel meets quarterly to provide input on issues brought to them by the PhD students. The members are voluntary but receive a voucher to acknowledge their time and associated costs attending. The panel had been meeting for a year prior to providing input on this study. The meeting at which this study was discussed took place at the Royal College of Surgeons in Ireland, lasted 2 hours in total including a break and was facilitated by BK and 2 other PhD students on the multimorbidity PhD program. There was one hour to discuss issues related to this trial with them and so their input was sought on three areas only outlined below.  For this study BK asked the panel to read the patient information leaflet (PIL) and comment on their understanding of the link worker intervention. They were asked how best to phrase the reason they had been invited by their GP to be part of the study. They also provided feedback on the wording and layout of the patient information leaflet. Finally they helped to co-design a brochure that summarised the study that could be distributed with the PIL. The overall results of the PhD (including a systematic review, this study and preliminary results of the process) evaluation were also presented to them and they were invited to provide general feedback as well as comment on potential target populations, intervention duration and how best to recruit to a similar study in future. |
| **3: Study results** |
| The PPI advisory group reviewed the patient information leaflet and made suggestions to improve it including reordering of sections to prioritise information about the intervention, adjusting language, and reducing repetition. They gave feedback on what they felt the essential information was and contributed to a brochure summarising this. Their opinion was sought on informing patients why they had been chosen to take part in the research. They approved the wording in the leaflet informing people that they had been invited due to having two or more ongoing health conditions and that their GP felt they may benefit from meeting the link worker.  They felt it was important to maintain flexibility in duration of the intervention as some people may benefit from shorter interventions and people living with multimorbidity can have many other commitments. They thought the ICECAP-A was a reasonable outcome measure. In future they thought recruitment during routine reviews would be acceptable. |
| **4: Discussion and conclusions** |
| The input of this group led to changes to recruitment materials which it was hoped would lead to a better understanding of the intervention and enhance recruitment. Notably the PPI group felt the term “multimorbidity” and “chronic condition” were negative and recommended removing these from any literature. They also felt that the information leaflets were overly cautious about an intervention they viewed as low risk and read more like the small print in an insurance policy rather than a research study.  The group unprompted brought up changing the name. Ultimately, the researchers did not act on the groups suggestion to rename as the link worker concept was already familiar to many GPs and community resource providers. |
| **5: Reflections/critical perspective** |
| The PPI group gave useful feedback on the recruitment material, but it was not possible to objectively test the impact this has on recruitment. The PPI group composition differed slightly from that of the target group for the intervention in that they came from all socioeconomic backgrounds. However, all members of the PPI group had experience of multimorbidity. The group have developed a relationship with the researcher over time and this allowed for a frank and productive conversation. Ultimately it would have been preferable to get the PPI group input prior to the pilot and again afterwards, but research team time constraints and capacity issues for the group, as they advise on 3 other projects, meant this was not possible. |
