# Supplementary material for: An exploratory randomised trial investigating feasibility, potential impact and cost effectiveness of link workers for people living with multimorbidity attending general practices in deprived urban communities
Source: BMC Prim Care. 2024 Jun 28;25:233. doi: 10.1186/s12875-024-02482-6 (PMC11212363; doi:10.1186/s12875-024-02482-6)
Supplement: Supplementary file 5 — Supplementary Material 5. [file 12875_2024_2482_MOESM5_ESM.docx]

| Supplementary Table 3. GP demographics, selection and recruitment rates | | | | | | | |
| --- | --- | --- | --- | --- | --- | --- | --- |
| ID | **Patients* living in deprived area** | **GMS list size** | **Patients on 5+meds** | **Patients deemed suitable for LW** | **Patients who were sent packs (from GP records)†** | **Recruited** | **Recruitment rate** |
|  |  | **n** | **n (%)** | **n (%)** | **n** | **n** | **%** |
| GP01 | 34% | 3865 | 784 (20%) | 538 (69%) | 85 | 21 | 25% |
| GP02a | 68% | 2136 | 140(7%) | 90 (64%) | 70 | 17 | 24% |
| GP02b | 73% | 2000 | 618 (31%) | 61 (10%) | 9 | 7 | 78% |
| GP03 | 39% | 2676 | 1459 (55%) | 270(19%) | 105 | 32 | 30% |
| GP04 | 77% | 4468 | 568 (13%) | 110 (19%) | 110 | 12 | 11% |
| GP05 | 45% | 2720 | 178 (7%) | 100 (56%) | 67 | 22 | 33% |
| GP06 | 37% | 1264 | *329 (26%)^1^* | *102(31%)^1^* | Missing data | 34 | Missing data |
| GP07 (A&B) | Missing data | 2285 | 1127 (49%) | 350 (31%) | 50 | 14 | 28% |
| GP08 | 24% | 3763 | 1030 (27%) | 142 (14%) | 30 | 14 | 47% |
| GP09 | 53% | 3400 | 744(22%) | 198 (27%) | 136 | 35 | 26% |
| GP10 | 88% | 4510 | 774 (17%) | 112 (14%) | 85 | 19 | 22% |
| GP11 | 28% | 1544 | 533 (35%) | 70 (13%) | 27 | 13 | 48% |
| Total eligible patients (estimate) |  |  |  | 2103 |  |  |  |
| *All registered patients. GMS: General Medical Services. † Due to recruitment challenges there was variation in approaches both between practices and over time, with some practices phoning patients in advance and only sending recruitment packs to patients who said they would be interested. Documentation of this was incomplete.^1^ Missing data was not available from one GP practice. To estimate the total number of eligible patients the mean proportion of patients on 5+ meds and deemed suitable for the intervention were calculated from the other practices | | | | | | | |

Supplementary File 5. Additional demographic, recruitment and analysis tables.

| **Supplementary Table 4. Demographic characteristics of those lost to follow up** | | | |
| --- | --- | --- | --- |
| **Demographic Characteristic** | **Complete data** | **Lost to follow up** | **P** |
| **Age >65** | 42% | 36% | 0.54 |
| **Female** | 64% | 70% | 0.53 |
| **Primary Ed** | 24% | 42% | 0.03 |
| **GMS** | 76% | 82% | 0.44 |
| **Employed** | 12% | 12% | 0.99 |
| **Housing (owner occupied)** | 49% | 42% | 0.48 |
| **Living alone** | 28% | 30% | 0.81 |
| **Other language** | 5% | 10% | 0.27 |
| **Smoker** | 32% | 49% | 0.06 |
| **Alcohol >11units** | 28% | 27% | 0.9 |

| **Supplementary Table 5 Demographics and baseline score differences between those who engaged and did not engage with link worker** | | | | |
| --- | --- | --- | --- | --- |
| **Column1** | **All Intervention**  **N=123** | **Met linkworker**  **N=102** | **Did not meet linkworker**  **N=21** | **p** |
| **Demographic characteristics** | n (%) | n (%) | n (%) |  |
| **Age Group** |  |  |  |  |
| 18-24 | 2 (2) | 2 (2) | 0 |  |
| 25-44 | 15 (12) | 12 ( 12) | 3 (14) |  |
| 45-64 | 53 (43) | 43 (42) | 10 (48) |  |
| 65+ | 53 (43) | 45 ( 44) | 8 (38) | 0.86 |
| Female | 78 (65) | 63 (62) | 15 (71) | 0.40 |
| GMS | 101 (87) | 82 (80) | 19 (91) | 0.27 |
| Meds 10+ | 73 (59) | 56 (55) | 17 (80) | 0.03 |
| Primary education or below | 35 (30) | 28 (28) | 7 (33) | 0.59 |
| Employed | 19 (18) | 16 (16) | 3 (14) | 0.87 |
| Home-owner occupied | 55 (50) | 46 (45) | 9 (43) | 0.85 |
| Living alone | 32 (27) | 26 (26) | 6 (29) | 0.77 |
| Other language | 9 (8) | 8 (8) | 1 (6) | 0.72 |
| Smoker | 40 (34) | 33 (32) | 11 (52) | 0.08 |
| Alcohol 11 units+ | 19 (16) | 31 (30) | 5 (24) | 0.55 |
| **PROM at baseline** | **Mean (SD)** | **Mean (SD)** | **Mean (SD)** | **p (mean diff 95% CI)** |
| EQ-5D-5l index | 0.473 (0.419) | 0.496 (0.388) | 0.361 (0 .549) | 0.89 (-.34 .074) |
| EQ-VAS | 60 (20.3) | 59.6 (21.0) | 61.5 (16.6) | 0.36 (-8.0 11.8) |
| HADS | 17.6 (8.9) | 17.3 ( 8.7 ) | 19.6 ( 10.0 ) | 0.15 ( -2.31 7.09) |
| HADS Anxiety | 9.6 (5.0) | 9.3 (4.7) | 11.3 (5.9) | 0.06 ( -.50 4.57) |
| HADS Depression | 7.8 (4.7) | 7.6 (4.7) | 8.3 (5.1) | 0.29 (-1.68 2.95) |
| ICECAP-A | 0.72 (0 .20) | 0.726 (0.204) | 0.706 (0.196) | 0.65 (-.12 .08) |
| PAM | 53.7 (13) | 53.8 (13.3) | 53.7 (12.4) | 0.51 (-6.47 6.35) |
| MM Treatment Burden | 20 (20) | 19.6 (19.0) | 21.9 (24.5) | 0.32 (-.30 .489) |
| Frenchay Activity | 41 (8.9) | 41.3 (8.6) | 39.7 (10.7) | 0.74 (-6.53 3.33) |
| Comparison of proportions using Pearson’s chi squared or comparison of means using t test | | | | |

| Supplementary Table 6: Planned subgroup analyses | | | |
| --- | --- | --- | --- |
| Subgroup | PROM | Mean Difference  (95% Confidence Interval) | p-value |
| Age ≤ 65 | EQ-5D-5L | -0.01 (-0.11 to 0.09) | 0.82 |
|  | HADS Anxiety | -0.26 (-1.32 to 0.80) | 0.62 |
|  | HADS Depression | -0.11 (-1.13 to 0.92) | 0.84 |
|  | ICECAP-A | 0.03 (-0.02 to 0.08) | 0.20 |
| Age >65 | EQ-5D-5L | 0.004 (-0.12 to 0.12) | 0.95 |
|  | HADS Anxiety | 0.19 (-1.03 to 1.42) | 0.76 |
|  | HADS Depression | 0.37 (-0.89 to 0.92) | 0.57 |
|  | ICECAP-A | 0.03 (-0.03 to 0.09) | 0.29 |
| Gender - Male | EQ-5D-5L | 0.003 (-0.12 to 0.13) | 0.96 |
|  | HADS Anxiety | -0.44 (-1.7 to 0.87) | 0.78 |
|  | HADS Depression | 0.17 (-1.14 to 1.49) | 0.80 |
|  | ICECAP-A | 0.07 (0.02 to 0.13) | 0.01 |
| Gender - Female | EQ-5D-5L | -0.01 (-0.11 to 0.09) | 0.83 |
|  | HADS Anxiety | 0.14 (-0.87 to 1.16) | 0.51 |
|  | HADS Depression | 0.03 (-0.97 to 1.03) | 0.96 |
|  | ICECAP-A | 0.003 (-0.04 to 0.04) | 0.89 |
| Complete case analysis using mixed regression model | | | |

| Supplementary Table 7: Per protocol analysis | | |
| --- | --- | --- |
| Outcome Measure | **Mean Difference (95% Confidence Interval)** | **p value** |
| EQ-5D-5L | 0.20 (-0.06 to 0.10) | 0.62 |
| EQ-VAS | 4.19 (-0.69 to 9.08) | 0.09 |
| HADS | 0.39 (-1.07 to 1.82) | 0.61 |
| HADS-Anxiety | 0.19 (-0.62 to 0.99) | 0.65 |
| HADS- Depression | 0.30 (-0.50 to 1.10) | 0.46 |
| ICECAP-A | 0.03 (-0.01 to 0.07) | 0.08 |
| PAM | 1.21 (-2.38 to 0.08) | 0.51 |
| MM Treatment Burden | -0.07 (-0.24 to 0.08) | 0.34 |
| Frenchay Activity Index | 2.10 (0.12 to 4.08) | 0.04 |
| Complete case analysis using mixed regression model comparing those who met the link worker at least once to control | | |

| **Supplementary Table 8a: Incremental Cost Effectiveness Results – Multivariate ICECAP-A – Sensitivity analysis –Full capacity and low costs based on RCSI salaries with reduced GP supports and LWs at full capacity** | | | | | | |
| --- | --- | --- | --- | --- | --- | --- |
| **Variable/ Analysis** | | | **Incremental Analysis**  (Intervention minus Control) | | | |
| **Cost Analysis** | | |  | | | |
|  | | | **Intervention** | | **Control** | |
| Mean Total Cost (SD) | | | €597.48 (245.48) | | €225.20 (735.34) | |
| Difference in Mean Total Cost €  (95% CIs)  [p-value] | | | €401.82  (210.06, 593.58)  [P=0.000] | | | |
| **Health Outcome Analysis** | | |  | | | |
|  | | | **Intervention** | | **Control** | |
| Mean QALYS (SD) | | | 0.720 (0.184) | | 0.693(0.186) | |
| Difference in Mean QALYs  (95% CIs)  [p-value] | | | 0.015  (-0.005, 0.035)  [p=0.852] | | | |
| ICER (Difference in cost/difference in QALY) | | | €26,855 | | | |
| ***Probability (%) that the Intervention is Cost Effective for Threshold Value (λ)*** | | | | | | |
| **λ = €0** | **λ = €5,000** | **λ = €10,000** | **λ = €20,000** | **λ = €30,000** | | **λ = €45,000** |
| **0.058** | **0.062** | **0.088** | **0.364** | **0.621** | | **0.787** |
| Cost analyses: GLM regression with log link function, Gamma variance function, estimated controlling for treatment group, baseline cost and General Practitioner clustering.  QALYs Analyses: GLM regression model, with identity link function, Gaussian variance function, estimated controlling for treatment group, baseline ICECAP- A and General Practitioner clustering.  Expected Cost Effectiveness Analysis: Probabilities estimated using nonparametric bootstrapping technique based on 1000 bootstrapped resamples | | | | | | |

| **Supplementary Table 8b: Incremental Cost Effectiveness Results – Multivariate ICECAP-A – Sensitivity analysis full capacity based on HSE LW salaries, reduced GP supports and link workers at full capacity** | | | | | | |
| --- | --- | --- | --- | --- | --- | --- |
| **Variable/ Analysis** | | | **Incremental Analysis**  (Intervention minus Control) | | | |
| **Cost Analysis** | | |  | | | |
|  | | | **Intervention** | | **Control** | |
| Mean Total Cost (SD) | | | €873.48 (245.48) | | €225.20 (735.34) | |
| Difference in Mean Total Cost €  (95% CIs)  [p-value] | | | €751.13  (495.89, 1,006.38,)  [P=0.000] | | | |
| **Health Outcome Analysis** | | |  | | | |
|  | | | **Intervention** | | **Control** | |
| Mean QALYS (SD) | | | 0.720 (0.184) | | 0.693(0.186) | |
| Difference in Mean QALYs  (95% CIs)  [p-value] | | | 0.015  (-0.005, 0.035)  [p=0.852] | | | |
| ICER (Difference in cost/difference in QALY) | | | €50,075 | | | |
| ***Probability (%) that the Intervention is Cost Effective for Threshold Value (λ)*** | | | | | | |
| **λ = €0** | **λ = €5,000** | **λ = €10,000** | **λ = €20,000** | **λ = €30,000** | | **λ = €45,000** |
| **0.058** | **0.058** | **0.058** | **0.100** | **0.272** | | **0.553** |
| Cost analyses: GLM regression with log link function, Gamma variance function, estimated controlling for treatment group, baseline cost and General Practitioner clustering.  QALYs Analyses: GLM regression model, with identity link function, Gaussian variance function, estimated controlling for treatment group, baseline ICECAP- A and General Practitioner clustering.  Expected Cost Effectiveness Analysis: Probabilities estimated using nonparametric bootstrapping technique based on 1000 bootstrapped resamples | | | | | | |

| **Supplementary Tale 9: Breakdown of Health Care Costs** | | | | |
| --- | --- | --- | --- | --- |
|  | **Intervention** | **Control** | **Intervention** | **Control** |
|  | **Baseline**  ***Mean(SD)*** | | **Follow Up**  ***Mean(SD)*** | |
| **Resource Items** |  |  |  |  |
| **GP Visits** | 70.07 (79.10) | 60.67 ( 63.75) | 58.61 (61.39) | 37.40 ( 47.68) |
| **PN Visits** | 11.81 (20.13) | 13.79 (24.87) | 6.05 (14.81) | 14.00 (28.85) |
| **OOH Visits** | 1.41 (7.56) | 2.92 (15.38) | 2.11 (10.67) | 3.28 (11.27) |
| **A&E Visits** | 7.34 (45.68 ) | 20.28 ( 74.14) | 12.24 (58.46) | 25.35 (82.11) |
| **Outpatient Visits** | 46.27 (102.00) | 45.4386 ( 88.33) | 39.15 (72.95) | 27.01 (61.42) |
| **Hospital days** | 130.44 (852.27) | 238.37 (1249.88) | 86.56 (940.27) | 118.14 ( 654.34) |
| **Total Healthcare cost Euro €** | Mean 267.35 (906.37)  Total- 31547.52  SE 9845.673  (95% CI 12147.83 - 50947.21) | Mean 379.91 (1315.41)  Total 42550.04  SE 13921.02  (95% CI 15120.37 -  69979.71) | Mean 134.48 (245.48)  Total 15868.78  SE 2666.592  (95% CI 10614.59 - 21122.97) | Mean 225.20 (735.34 )  Total- 25532.67  SE 7848.16  (95% CI 10068.83 - 40996.51) |
| GP – general practitioner PN – Practice Nurse OOH – Out of Hours A&E – Accident and Emergency  Completeness of data:  **Intervention**: *Baseline –* 4% missing data for HCU,8% EQ-5D-5L. 7% ICECAP-A  **Control**: *Baseline* – 2% missing data for HCU, 8% on EQ-5D-5L. 6% ICECAP-A  **Intervention**: *Follow-up (Time 2) –* 4% missing data for HCU, 18..% for EQ-5D-5L. 17% for ICECAP-A  **Control**: *Follow-up (Time 2)*  – <1% missing data for HCU, 12% on EQ-5D-5L. 16% ICECAP-A  Where necessary unit costs were inflated using the health component of the consumer price index from the Central Statistics office^38^ | | | | |
